# IDSSR: An Efficient Pipeline for Identifying Polymorphic Microsatellites from a Single Genome Sequence

**DOI:** 10.3390/ijms20143497

**Published:** 2019-07-16

**Authors:** Xuan-Min Guang, Jin-Quan Xia, Jian-Qing Lin, Jun Yu, Qiu-Hong Wan, Sheng-Guo Fang

**Affiliations:** MOE Key Laboratory of Biosystems Homeostasis & Protection, State Conservation Centre for Gene Resources of Endangered Wildlife, College of Life Sciences, Zhejiang University, Hangzhou 310058, China

**Keywords:** polymorphic SSRs, INDEL, IDSSR, efficient, high quality

## Abstract

Simple sequence repeats (SSRs) are known as microsatellites, and consist of tandem 1–6-base motifs. They have become one of the most popular molecular markers, and are widely used in molecular ecology, conservation biology, molecular breeding, and many other fields. Previously reported methods identify monomorphic and polymorphic SSRs and determine the polymorphic SSRs via experimental validation, which is potentially time-consuming and costly. Herein, we present a new strategy named insertion/deletion (INDEL) SSR (IDSSR) to identify polymorphic SSRs by integrating SSRs with nucleotide insertions/deletions (INDEL) solely based on a single genome sequence and the sequenced pair-end reads. These INDEL indexes and polymorphic SSRs were identified, as well as the number of repeats, repeat motifs, chromosome location, annealing temperature, and primer sequences, enabling future experimental approaches to determine the correctness and polymorphism. Experimental validation with the giant panda demonstrated that our method has high reliability and stability. The efficient SSR pipeline would help researchers obtain high-quality genetic markers for plants and animals of interest, save labor, and reduce costly marker-screening experiments. IDSSR is freely available at https://github.com/Allsummerking/IDSSR.

## 1. Introduction

Simple sequence repeats (SSRs), also known as microsatellites, are tandem 1–6-bp motifs distributed frequently throughout eukaryote genomes [1]. Currently, microsatellites are one of the most popular molecular markers, owing to their advantages including high polymorphism, co-dominant inheritance, and reproducibility in different in vitro conditions [2]. Furthermore, they have been widely used in studies on molecular ecology [3], conservation biology [4], molecular breeding [5], and many other fields. Nevertheless, the major limitation of microsatellites is the need to isolate them de novo from the species under study. Furthermore, traditional microsatellite isolation strategies based on clone and probe hybridization are not only costly and labor- and time-intensive, but also inefficient [6].

With the development of next-generation sequencing (NSG), genome sequencing has become faster and cheaper, enabling the sequencing of hundreds of genomes and transcriptomes of important organisms. Sequence technology has changed how microsatellite markers are identified using genome sequencing and bioinformatics tools [7,8,9]. To date, more than 25 tools or methods are available for identifying microsatellites from genome or RNA sequences, and this number is increasing [10,11,12]. These tools, such as Tandem Repeat Finder (TRF) [13], GMATo [14], SSRIT [15], SSR-pipeline [16], MREPS [17], PRoGeRF [18], MISA [19], Kmer-SSR [20], ESAP plus [21], SA-SRR [22], PERF [23], and SciRoko [24], are used to conduct SSR mining. In contrast, other tools such as SSRLocator [25], QDD [26], CandiSRR [27], GMATA [28], and SSRPoly [29] have been improvised with the inclusion of a primer design algorithm. Most of these methods are focused on identifying the SSR itself, and some have integrated the tools for identifying SSRs with primer design.

Despite the existence of numerous tools, the availability of efficient tools or pipelines for identifying candidate SSRs without false positive results and limited quality is still an issue, arising partly because the identified SSRs may be monomorphic or their proximal primers are not specific, thus potentially resulting in homoplasy. Further experiments are often required to identify the polymorphic SSRs and finally validate the data. Obviously, the disadvantages of monomorphic SSRs or low quality of their primers will greatly affect the subsequent experiments, such as PCR, leading to wastage of energy and financial resources.

In addition to SSR markers, nucleotide insertion/deletions (INDELs) are one of the most abundant structural variants, widely distributed across the genomes of plants and animals. INDELs can be distinguished easily based on their sizes, and, because of their moderate polymorphism, they can be amplified using conventional PCR and direct gel electrophoresis methods. These characteristics have made INDELs highly valuable for identifying other markers such as SSRs and single-nucleotide polymorphisms (SNPs), and they can be used as effective markers in genetic analysis [30,31,32]. Moreover, INDEL markers are easily detectable in genome and transcript sequences based on different bioinformatics tools.

SSRs and INDELs are both extremely useful, and, therefore, it would be expedient to develop a method that integrates these two types of markers for identifying highly polymorphic SSRs. Herein, we present a new protocol that integrates SSRs and INDEL markers to identify polymorphic SSRs and high-quality specific primers, which exhibit good performance, repeatability, strong stability, and an extremely low genotyping error rate based solely on a single genome sequence. The strategy identifies SSRs from genome sequences using the improved SSRIT tools, designs the primers for each SSR, filters the low-quality primers and their SSRs, and combines the high-quality SSRs with the INDEL markers throughout the genome to generate dependable microsatellite results with polymorphisms. We used this pipeline to identify microsatellites on the giant panda reference genome, and investigated the efficacy and applicability of these markers in a wild population, thus demonstrating the impressive capacity of our pipeline. This highly efficient SSR pipeline is expected to facilitate subsequent genetic analyses of plants and animals of interest and save time, labor, and the cost associated with marker screening experiments.

## 2. Results

### 2.1. Identification of Candidate Polymorphic SSRs in Giant Panda, Gallus gallus, and other Assemblies

The giant panda is a critically endangered species in China. Currently, although some SSRs have been identified in the giant panda, obtaining microsatellites with tri-, tetra-, and pentanucleotides with high polymorphism remains challenging [33,34,35,36,37,38,39]. In this study, we identified polymorphic SSRs in the giant panda using our new pipeline.

First, 267,958 INDELS of 1–6 bp were identified (Table S1). Among them, 189,236 deletions and 78,722 insertions were found throughout the whole genome. The genome-wide average density was about 0.12 INDELS per Mb. The number of deletions and insertions had a relatively regular distribution: Insertions and deletions with 1 bp in length occurred the most often, and short INDELs were more abundant than long ones (Figure S1). The INDEL rate was 1.2 × 10^−4^ on autosomes and 0.7 × 10^−4^ on sex chromosomes. Thereafter, 423,459 SSRs with high-quality primers were identified, with a total length of 7.2 Mb. Among them, dinucleotide repeats were the most abundant (225,439, 53%), followed by tetranucleotide repeats (87,106, 20.6%), trinucleotide repeats (92,099, 21.7%), pentanucleotide repeats (16,961, 4%), and hexanucleotide repeats (1854, 0.4%). The number of each type is comparable with the previously reported results on the giant panda [33]. Among these SSRs, CT/GA (26.4%) and AC/GT (21.6%) were quite dominant, and TTTA/AAAT (5.7%) was the most frequent motif for >2 SSR units. Moreover, it was found that SSRs were more abundant in intergenic regions (297,202 SSRs) than in the gene regions (126,257 SSRs). To identify polymorphic SSRs, those in an INDEL variation region were selected as final candidate SSRs. Consequently, 4882 polymorphic SSRs with an average length of 18 bp and average repetitions of 8.58 were detected in the giant panda genome. This total number of SSRs is much less than previously reported in the giant panda. These SSRs were present in 1793 scaffolds, with a relative abundance of 2 SSRs/Mb (Table S2), and this density is significantly lower than the previous value of 372 SSRs/Mb [33]. Among the 4882 polymorphic SSRs, the dinucleotide repeat motif was the most abundant (>83%), with motifs (GA)n and (AC)n the most frequent SSRs, consistent with previous reports on the giant panda [33]. Furthermore, the frequencies of the trinucleotide and tetranucleotide motifs were similar at 8.7% and 7.4%, respectively (Table 1). Those ratios are very different from the previous reports of 4.2% for trinucleotides and 18.09% for tetranucleotides, respectively. We identified a limited number of pentanucleotide repeats (26) and only one hexanucleotide repeat. The total number of repeat motif types was 241, and there were five most-abundant motif repeat classes, including (GA/TC)n, (AC/GT)n, (AT)n, (AGAG)n, and (ACAC)n. The frequency of di-nucleotide repeats is consistent with previous reports but different from the other three motif units. In total, the aforementioned five classes of repeats constituted 80% of all the identified polymorphic SSRs, while the other >200 types only accounted for 20% (Figure S2).

**Figure 1:**
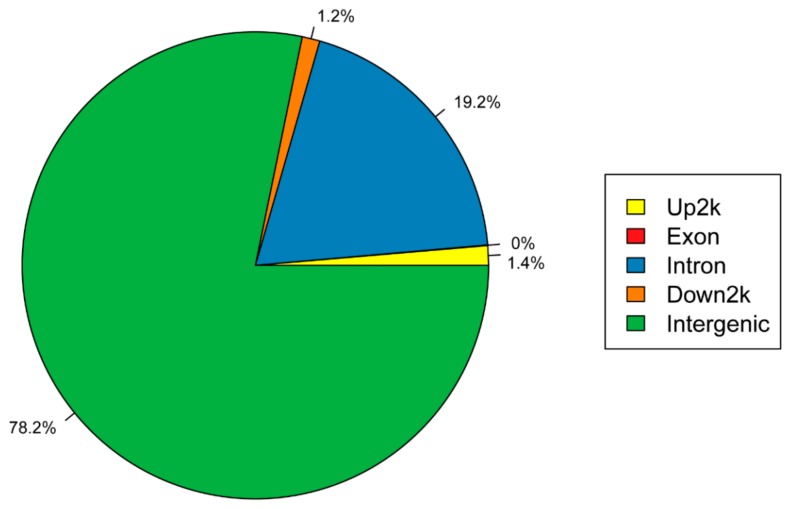
Locations of simple sequence repeats in the giant panda genome.

To investigate the distribution of the polymorphic SSRs across the genome, the polymorphic SSRs were annotated on the basis of the annotation data of the giant panda [40]. The entire distribution is shown in Figure 1. Most SSRs were in the intergenic regions (3819 SSRs), followed by polymorphic SSRs in introns (935 SSRs), and the rest were present 2 kb upstream or downstream of proximal genes. This distribution in the giant panda is somewhat different from that reported previously, albeit similar to that in bovid genomes [41].

In order to have a further validation of the strengths of our pipeline, genome assembly of *Gallus gallus* and other assemblies were used. We identified 2157 SSRs throughout the whole *Gallus gallus* genome (Table S3). Results showed that the genome-wide average density of SSRs in *Gallus gallus* was about 1.9 per Mb, which is nearly the same as in the giant panda. Among these SSRs, there are 1388 SSRs in intergenic regions, which is almost 100% higher than in gene regions (769). Among the 769 SSRs, only 91 were found in the exonic region. Interestingly, tetranucleotide SSRs were the most frequent unit, then followed by the pattern: Tri- > di- > penta- > hexanucleotide SSRs in the *Gallus gallus* genome (Table 1). It was found that (AT/TA)n, (AAC/TTG)n, and (TG/AC)n were the three most dominant repeat motifs. For those download assemblies, MISA-web [42] was used for predicting SSRs. We identified 117 SSRs, including 62 mononucleotide SSRs and 55 polyribonucleotide SSRs. Based on our pipeline, we identified 106 polyribonucleotide SSRs and 44 SSRs (80%), which were also identified through the MISA-web.

### 2.2. Evaluation of Microsatellite Polymorphism

To quantify the accuracy of those polymorphic SSRs detected using the pipeline, we analyzed the 26 microsatellite loci with 5-bp repeats and the single microsatellite locus with a 6-bp repeat for PCR experiments. Most of the primers (21 pairs, 77.78%), including the single 6-bp repeat, were successfully amplified and showed good performance, with specific amplification and high yield (Table 2).

In addition, genotyping was carried out to evaluate the polymorphism using 21 fluorescence-labeled microsatellite primers. The results show that all these loci were polymorphic (Table S4). From the 20 individual giant pandas, we acquired 2.38 alleles per locus on average, ranging from two to four, indicating the effectiveness of the pipeline in identifying polymorphic microsatellite loci and designing primers for microsatellite genotyping. These validated results reveal 100% polymorphic SSRs in the giant panda genome.

## 3. Discussion

In the present study, the protocol was developed for identifying polymorphic SSRs. In order to evaluate its efficiency, we applied our protocol to identify SSRs in the giant panda genome, *Gallus gallus*, and other assemblies. Compared with the previous results on the giant panda [33], we identified markedly fewer SSRs, with a markedly lower relative density. This phenomenon occurred primarily because our pipeline disregarded mononucleotide motifs, which constitute one of the most diverse SSRs in species. Moreover, the criterion that motifs should have unique primers and SSRs within an INDEL allele index markedly decreased the number of polymorphic SSRs. This rigorous filtering decreased the number of SSRs as well as the types identified, which makes a direct comparison with previous research difficult. Despite the significant differences between our final SSRs and those previously reported, we note that the intermediate results could be compared with previous studies. The entire distribution of SSRs in the giant panda genome is consistent with previous reports, showing that the occurrence of SSRs is lower in gene regions than in other regions because SSRs have a high mutation rate that potentially affects gene expression. To further verify the capacity of the pipeline, we applied the pipeline on the *Gallus gallus* genome and several other assemblies. The identified SSRs in *Gallus gallus* demonstrate that the pipeline also has utility on a more-studied species. Results from the other assemblies showed that our pipeline has high confidence, with the MISA-web method also indicating that our pipeline has an accuracy of over 80%. Polymorphic SSRs with motifs longer than 4 bp are typically more biologically interesting and extremely valuable for endangered animals. Hence, we selected SSRs of >4 bp, identified herein, for subsequent analysis. Excluding the failure of six primers, the remaining 21 primers displayed 100% polymorphic SSRs in the giant panda genome and considerably outperformed those previously reported, with rates of 71.7% [34], 88.5% [33], and 80% [43]. These previous validations were carried out using 1–4-bp SSRs, but the present validation was conducted using >4 bp SSRs. Overall, the experimental validation of those SSRs in the giant panda genome demonstrated the high success rate and the potential of our pipeline.

While IDSSR could provide substantially superior performance in identifying polymorphic SSRs with unique primers, the pipeline has some limitations. First, since INDEL markers should be obtained in the first stage, some reads with a short insert size library should be downloaded or sequenced, and the data should approach 25× genome coverage. This may impede its use, as reference genome reads may not be available. Moreover, INDEL calling is only based on the de novo genome reads, which may underestimate their number in the population. In future studies, we could improve this by using re-sequenced data of additional individuals. Second, to obtain a unique primer pair for each polymorphic SSR, we incorporated some initial filtering criteria. Since genome complexities often differ, this protocol may be time- and data-intensive, largely depending on the BLAST alignment algorithm, thereby impeding the identification of high-quality primers and SSRs.

Notwithstanding previously reported tools or pipelines for identifying candidate polymorphic SSRs, some including primer design, there is still a major limitation in analyzing SSRs and designing high-quality primers from large sequences. For a diploid genome, the assembled genome sequence is always haploid. The two sets of the chromosomes contain SSRs with heterozygous INDELs, and individuals harbor SSRs that may be polymorphic among other samples in genetic analyses. Accordingly, we present the IDSSR pipeline to identify numerous efficient and polymorphic SSRs and their specific primers based on assembled and non-assembled genome sequencing reads. In the first stage of the pipeline, high-quality INDELs were identified in accordance with the reference genome data and clean reads. In the second stage, SSRs with specific primers were designed using several filtering steps, and finally, SSRs within a repeated motif as altered INDEL alleles were selected as the final output. As the altered INDEL allele is a repetition of the repeat motif, this ensures that the identified SSRs are polymorphic. This is the first study to use INDEL and SSR markers together to identify the abundance of polymorphic SSRs. This integration could theoretically improve the success rate of identifying polymorphic SSRs. The output of the pipeline revealed that the numbers of repeats, repeat motifs, repeat positions, SSRs, chromosome locations, annealing temperatures, and primer sequences were convenient for use in further analyses.

Overall, our novel pipeline developed herein would be a suitable supplementary tool for establishing high-quality SSRs, and would help resolve biological issues.

## 4. Materials and Methods

### 4.1. Data Accessibility

The genome sequences of the giant panda and *Gallus gallus* are available at the ENSEMBL Genomes Database (ftp://ftp.ensembl.org/pub/release-91/fasta/ailuropoda_melanoleuca/dna/, ftp://ftp.ensembl.org/pub/release-96/fasta/gallus_gallus/dna). The sequenced reads for the giant panda can be downloaded from the National Centre for Biotechnology Information (NCBI) Sequence Read Archive (SRA) under accession numbers SRX1352277 and SRX1352276. The sequenced reads for *Gallus gallus* can be downloaded with accession number SRR8902348. The assemblies (accession numbers: AC256511.1, AC269605.1, AC265197.1, AC263353.1, AC264961.1, AC266636.1, AC261250.1, AC267178.1, AC259365.1, AC257258.1) were also downloaded from the NCBI database.

### 4.2. Package Availability and Requirements

- Project name: IDSSR
- Project homepage: https://github.com/Allsummerking/IDSSR
- Operating system(s): Linux and UNIX
- Programming language or software: Perl, BASH, BLAST [44], Primer3 [45], and SSRIT [15].

### 4.3. Complete SSR Pipeline Process

**Figure 2:**
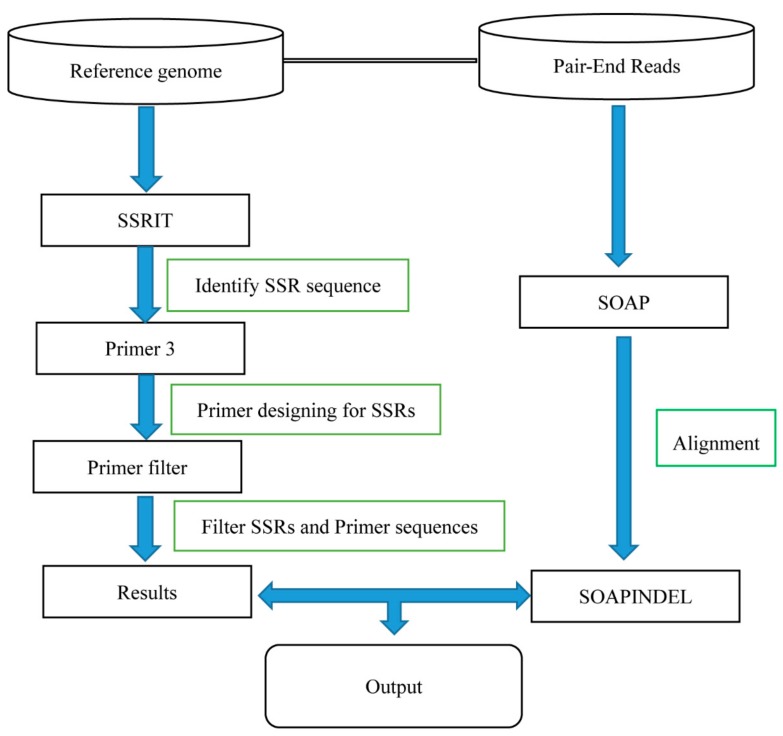
Flowchart of the insertion/deletion SSR (IDSSR) pipeline.

The input files for IDSSR were assembled genome sequences in the FASTA format and sequenced clean reads in the FASTQ format. All the procedures for identifying candidate SSRs using this pipeline can be separated into two parts: Calling INDELs and identifying SSRs (Figure 2).

INDEL calling involved downloading or assembling the genome of the target species in question. Thereafter, clean paired-end (PE) reads of the target species were prepared. All clean PE reads were aligned to the target genome sequence, using SOAP2 [46] with the following parameters: ‘-p 2 –m 170 –x 800 –s 32 –l 24 –v 30′. Alignments generated via PCR duplication were eliminated, and the mapped files were sorted in accordance with the mapping coordinates. The file was then used as an input for the SOAPindel [47] software based on the following parameters: ‘-m 1 –p 0.01 –c 3 –h 0.5 –k 5′ to identify INDELs. Then, the returned results were filtered as follows: (i) INDELs should have at least five supporting PE reads, and (ii) sites should be at least 5 bp away from their predicted neighboring INDELs. Finally, we obtained 1–6-bp high-quality INDELs throughout the genome.

The identification of SSRs with high-quality specific primers and the entire protocol can be divided into five steps: (1) Different 2–6-bp motifs were detected using an improved SSRIT [15], and this software paper was cited more than 1700 times and used to screen the whole genome. The number of repeats for a 2-bp motif should be >6-fold and >4 for the 3–6-bp motif. (2) Each candidate SSR sequence with 150 bp flanking sequences on both sides was imported into the software Primer 3 [45] for designing primers using the following parameter settings: The primer length should be approximately 20–28 bp, the optimal GC content is 40%–60%, the minimum and maximum annealing temperatures are 60 and 65 °C, and the product size has a range from 100 to 300 bp. (3) Those primers with any SSR motif were filtered initially, thus reducing homoplasy, and the rest of the primers were aligned to the reference genome, using BLASTn [44] for further filtering. According to the BLASTn results, those primers with a mismatch of >4 in the forward or one in the reverse against the genome were discarded. Furthermore, to obtain specific primers, only primers with only one hit, or multiple hits with products of different lengths of >2 kb were retained. (4) These product sequences were then searched to identify 2–6-bp motifs using the improved SSRIT with the same criterion used in the first step, and products with multiple SSRs were discarded. The best-fitting pairs of primer sequences and the proximal SSRs were retained. As the primers probably result in homoplasy, these rather strict filters may greatly improve the success ratio for amplifying primers. (5) The key step is that INDELs obtained initially were used to identify polymorphic SSRs. For all high-quality SSR motifs, whole INDELs were identified and compared with each SSR. INDELs were integrated into the SSRs based on the following criteria: a) The altered INDEL allele has the same repeat unit as the SSR motif, or the altered allele of the INDEL is precisely the SSR’s repeat motif; and b) the INDEL is located at the center of the SSR motif. Finally, SSRs containing INDELs were selected as candidate polymorphic SSRs.

The procedures above were implemented using the Perl and BASH scripts, and the whole process was developed to be user-friendly.

### 4.4. Sample Collection and DNA Extraction

To experimentally validate the polymorphic SSRs, the blood samples of giant pandas were collected by veterinarians in the China Research and Conservation Center for the Giant Panda during the routine physical examinations, and stored in the State Conservation Center for Gene Resources of Endangered Wildlife. The use of the samples was permitted by the State Conservation Center for Gene Resources of Endangered Wildlife (SCCGREW2016-S11) on 13 October 2016. The conventional phenol–chloroform method was used to extract the genomic DNA from the blood samples [48].

### 4.5. Evaluation of Microsatellite Polymorphisms

We carried out PCR with genomic DNA from the giant panda “Panpan” to evaluate the sensitivity and specificity of the primers, as well as the optimum annealing temperatures. The microsatellite primers used in this study are listed in Table 2. The details of the PCR process are as follows: 94 °C for 3 min, followed by 35 cycles at 94 °C for 30 s, 30 s at the annealing temperature, 72 °C for 30 s, and 72 °C for 10 min in the final extension step. Each PCR reaction mixture contained 5 μL 2× Taq master Mix (Shanghai Generay Biotech, Shanghai, China), 0.4 μL of each primer (10 μM), and 0.5 μg of genomic DNA in a total volume of 10 μL. PCR products were visualized on a 1% agarose gel.

Subsequently, 21 pairs of primers with high sensitivity and specificity were used for microsatellite polymorphism analysis. The fluorescence-labelled forward primers (tetrachloro-6-car-boxyfluorescein (TET), 6-carboxyfluorescein (FAM), and hexachloro-6-car-boxyfluorescein (HEX) dyes) were synthesized and used for the PCR with genomic DNA from 20 giant pandas. PCR products were diluted 1:10 and run using the 3730 DNA analyzer (Applied Biosystems, Foster City, CA, USA) with the GeneScan 500 ROX Size standard (Applied Biosystems). The output data were analyzed using Gene Mapper 4.1 (Applied Biosystems) to assign the genotype to each sample at each locus.

## Figures and Tables

**Table 1:** Distribution of polymorphic simple sequence repeats (SSRs) in the giant panda and *Gallus gallus*.

	SSR Length (bp)	Dinucleotide	Trinucleotide	Tetranucleotide	Pentanucleotide	Hexanucleotide	Total
Giant Panda	Number	4064	428	363	26	1	4882
	Length (bp)	75,324	6933	7204	580	24	90,065
	Percentage of repeat	83.24%	8.77%	7.44%	0.53%	0.02%	100%
*Gallus gallus*	Number	453	621	763	273	47	2157
	Length (bp)	9412	8067	19,176	8650	1482	46,787
	Percentage of repeat	21.00%	28.79%	35.37%	12.66%	2.17%	100%

**Table 2:** The primers of the giant panda microsatellites.

Locus	Number of Bases per Repeat Unit	Forward Primer Sequence	Reverse Primer Sequence	Label *
GP1	6	CTCGTGCTGGGCTGAAGAGAGAAG	CCCCATCACAATGTCTGCAGCTG	5′-TET
GP2	5	GATGGGCCACCTTGACATGTACAT	ACTGAAGACCCAGGAGAGAGCTTT	5′-FAM
GP3	5	AACAAAAACCCCCAAACCAAACCC	GGTCGGTAGCTATGAAGTGTTGGG	5′-FAM
GP4	5	TCATTGTTACTCTGCCTGTATCTGTT	CTTGTGCTCTCTCTCCGTCAAATA	--
GP5	5	ACCACAGCCAAGGGTTGTATTGTT	GGGTTGTGAGTTGAAGCCCTACAT	5′-FAM
GP6	5	CTCAAGGCAGTTGTTCCCACTCTT	TCCATATTGGAAAACCCTACACTGGAA	5′-FAM
GP7	5	TGGTGGTAATGAAATCCCTCAGCT	CTTCTATCCTCAGTGAAGCCGTCC	5′-TET
GP8	5	CTTACTTTCACATCTGGGCCCTCC	ACATGCAATGAAACAGGGACCACT	5′-TET
GP9	5	TTAACTGGGGGTGTACTGGATGGT	TAAGGGTGCTATTCTCGCCATTCC	5′-FAM
GP10	5	CTCGGAGGGCATCTGTTGGATTAA	CCATGAGCGTGGGGCCTATTTAAA	5′-FAM
GP11	5	TCTTCAACAAAACAATTCTTTTGCTTGT	TTAAAACCAGCGTGGCAGATTTTG	-
GP12	5	CCAACTCACGGAGGGGATATCAAG	AACCACATCCTATTCTGACTGCCT	-
GP13	5	CCTCAACTCCTTCCCCTGCAAAAT	GGTGTCGTCAAGTACATGGGTCTC	5′-TET
GP14	5	TCTGTCAGCTGAGTTGACCTTGAG	TTTGCAGCAAAAAGTTCTCTTGCC	-
GP15	5	GAGACAGGCTATCTTACATTGGGCT	AATTGTAGCAGGGTCTCATGGCTG	5′-HEX
GP16	5	TATCTCTAAGTGCCCTGGGGTCAG	CGGACTCGTTCCTAGTGTGTGG	5′-HEX
GP17	5	TCGTTGAACGCCACATCAAAAACT	TTCAGGATTCTGGGCACTACTGGA	5′-HEX
GP18	5	TCGAGGGCTTGCGACTTTATTTCA	AGAGCTGGATTGGAGAAAGCTTGA	5′-TET
GP19	5	AGGAAGGGAAGGGAAGGGAAAGAA	TCCTCACAAACCAGAGAGTATGGGA	-
GP20	5	TGCTCGAAAGGAAACTACCAGGAA	CCAAGGTCATGGAGGCACATTTTA	5′-HEX
GP21	5	ACAAATGCAATAGAAGGGAAAGTCTGT	ATGGTGCCCTGGGTGTTATACG	-
GP22	5	TTTGGAGAGGCGGAAAGAGCTTTT	TTTTGCTGCGAGGAGGTGATAGTC	5′-HEX
GP23	5	GGCGTCCCAGTACGTAACTCTCTA	ATACACTTTGGAGGCACCTGGATG	5′-TET
GP24	5	GATATTCTCTCTCCCTCTCCCCTG	TTCCATTTTGAGCCAAAAGTTACTTAGT	5′-TET
GP25	5	CATCTGAGCACTTGAAAGCCAGT	GTCACTACAGCAATCATATAACCCTGT	5′-HEX
GP26	5	CTCAGGATCGTGAGTTTAAGCCCC	GGTTGTCTTATTTCCTGTGCATTTGGT	5′-HEX
GP27	5	TCCAGCTAAACAAACTGCCCTTCT	CTACTGGTCAGCTGCAAGGACTTG	5′-TET

* TET: tetrachloro-6-car-boxyfluorescein; FAM: 6-carboxyfluorescein; HEX: hexachloro-6-car-boxyfluorescein.

## Supplementary Materials

Supplementary materials can be found at https://www.mdpi.com/1422-0067/20/14/3497/s1.
