# Supplementary material for: IDSSR: An Efficient Pipeline for Identifying Polymorphic Microsatellites from a Single Genome Sequence
Source: Int J Mol Sci. 2019 Jul 16;20(14):3497. doi: 10.3390/ijms20143497 (PMC6678329; doi:10.3390/ijms20143497)
Supplement: Supplementary file 1 [file ijms-20-03497-s001.zip › Figure S2.pdf]

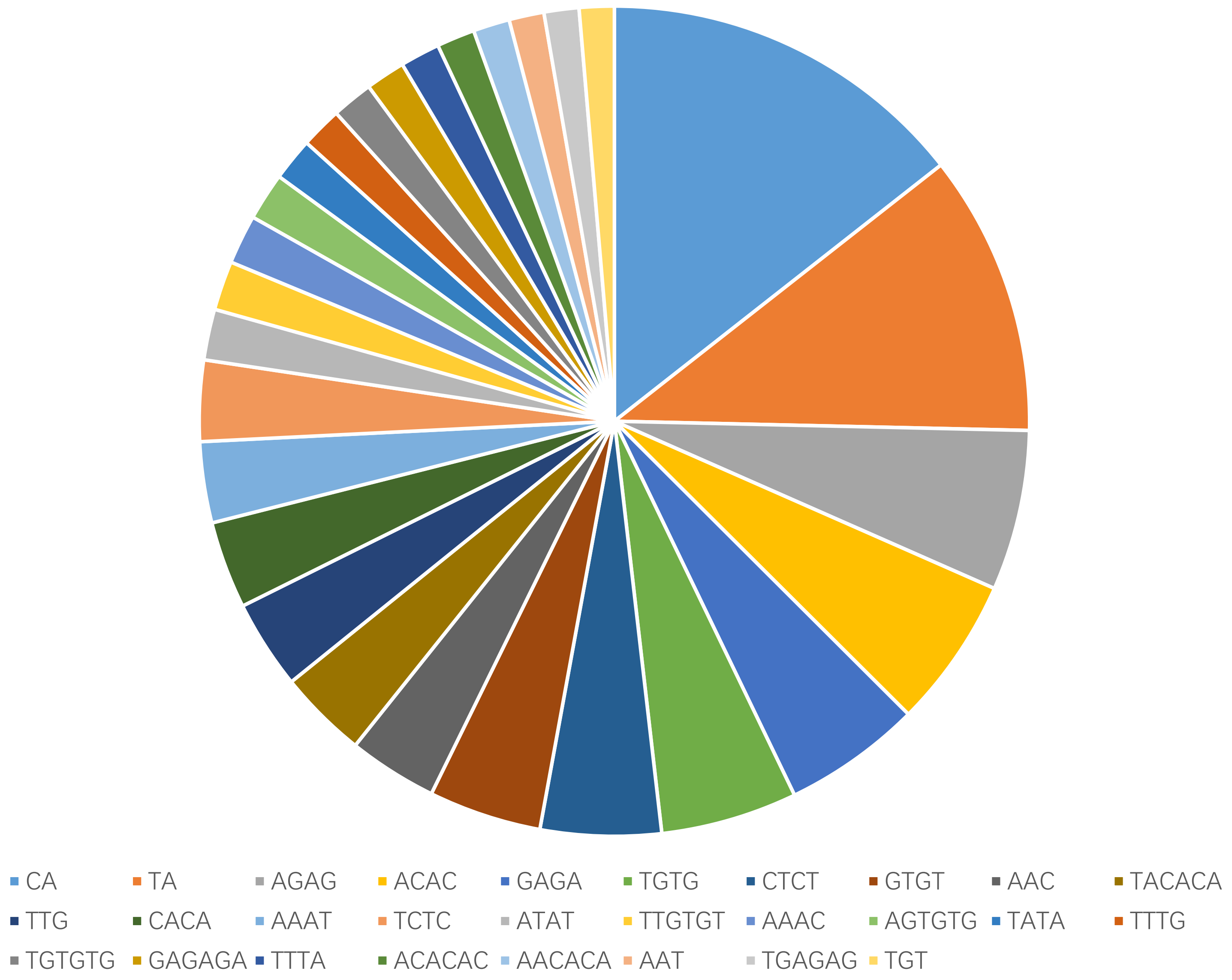
CA
TA
AGAG
ACAC
GAGA
TGTG
CTCT
GTGT
AAC
TACACA
TTG
CACA
AAAT
TCTC
ATAT
TTGTGT
AAAC
AGTGTG
TATA
TTTG
TGTGTG
GAGAGA
TTTA
ACACAC
AACACA
AAT
TGAGAG
TGT
