# Supplementary figures and images for: IDSSR: An Efficient Pipeline for Identifying Polymorphic Microsatellites from a Single Genome Sequence
Source: Int J Mol Sci. 2019 Jul 16;20(14):3497. doi: 10.3390/ijms20143497 (PMC6678329; doi:10.3390/ijms20143497)

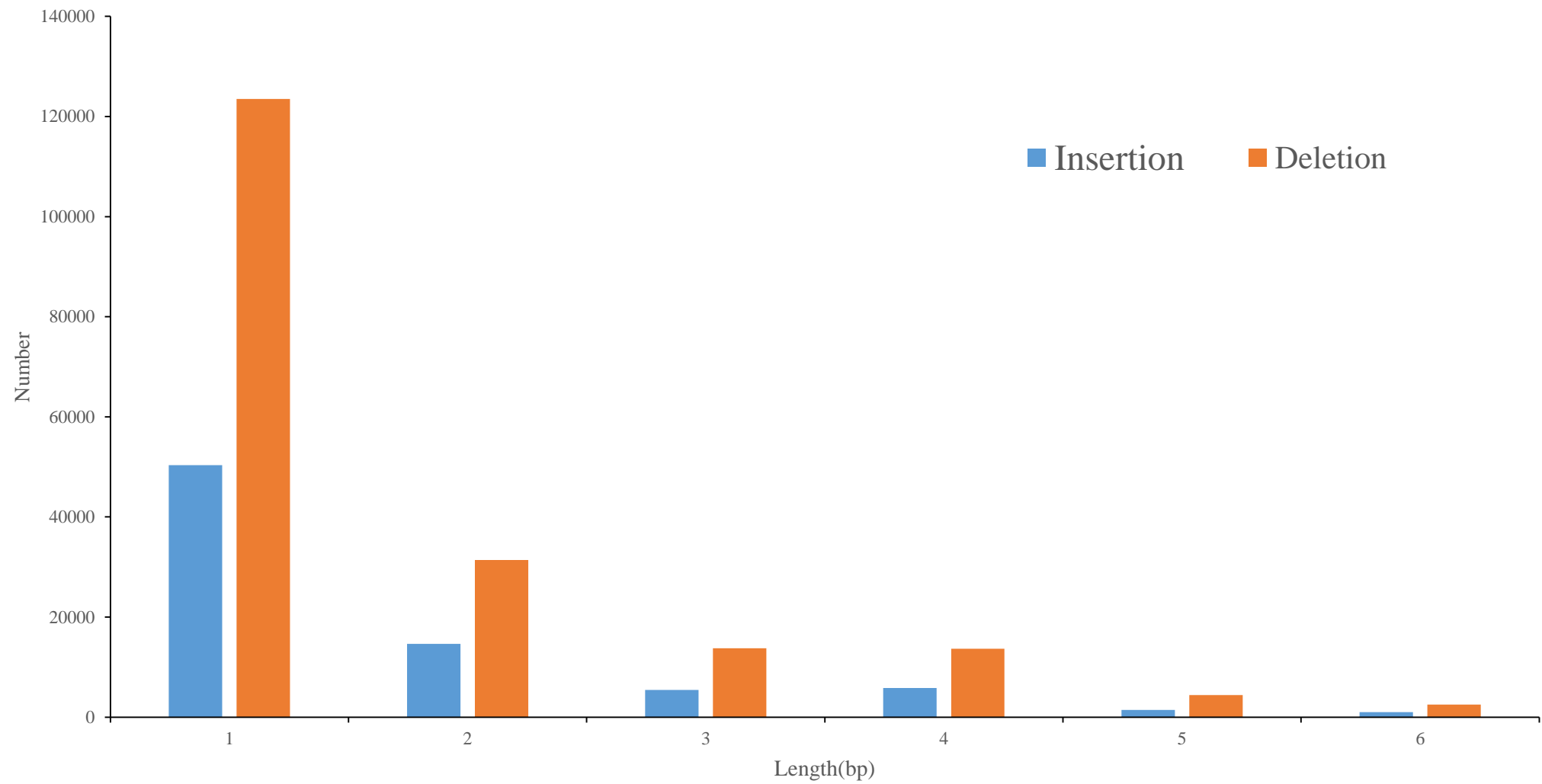

Supplement: Supplementary file 1 [file ijms-20-03497-s001.zip › Figure S1.pdf]
